# Investigating the effect of changing the substrate material analyzed by laser-induced breakdown spectroscopy on the antenna performance

**DOI:** 10.1038/s41598-024-52435-3

**Published:** 2024-01-23

**Authors:** Ashraf S. Abdel Halim, Zienab Abdel-Salam, Mohamed Abdel-Harith, Omnia Hamdy

**Affiliations:** 1 Department of Communication, Faculty of Engineering, Canadian International College (CIC), Cairo, Egypt; 2 Laser Applications in Metrology, Photochemistry, and Agriculture Department, National Institute of Laser Enhanced Science, Cairo University, Giza, Egypt; 3 Department of Engineering Applications of Lasers, National Institute of Laser Enhanced Sciences, Cairo University, Giza, 12613 Egypt

**Keywords:** Electrical and electronic engineering, Condensed-matter physics

## Abstract

Miniaturized microstrip antennas are efficiently utilized in MICS band wearable and implantable medical applications. However, the properties of the materials employed for antenna fabrication influence its resultant parameters and play a vital role in its performance. Rogers have been widely used as a substrate material in various antenna designs. In this work, a proof of concept study has been conducted to determine how altering the substrate used in antenna construction affects antenna performance. Using the laser-induced breakdown spectroscopy (LIBS) approach, the elements present in the two distinct substrate raw materials were compared to investigate potential effects on the antenna’s performance. Given their accessibility and widespread use, two types of Rogers’ substrates, RO 3210 and RO 4003, were selected. Furthermore, two identical antenna designs were modeled and fabricated using the two substrate materials. The reflection coefficient (S11) and other antenna parameters were determined and compared. Moreover, the recorded LIBS spectra were evaluated using principle component analysis and partial least square regression techniques. The LIBS spectra showed different copper and iron contents between the two Rogers (i.e., other dielectric properties), leading to a frequency shift. Additionally, impurities in the fabricated material increase the possible losses. Consequently, the elemental contents of the utilized Rogers control the antenna’s performance and can ensure its safety in wearable and implant applications.

## Introduction

Recently, antennas designed for wireless biotelemetry and telemedicine applications have attracted great interest owing to the progress in medical technology and microelectronics^1–3^. These antennas have been employed for wireless communication between certain human organs and an external device (such as a smartphone) for monitoring physiological parameters, including blood pressure, glucose level, and heartbeat^4^. Such parameters are essential for accurate diagnosis and effective therapy^5^. Accordingly, various antenna designs in the medical implant communication service (MICS) band have been proposed^6^. However, implantable antennas must contend with constraints such as size, operating frequency authorization, biocompatibility, and varying tissue dielectric characteristics, which impair antenna performance and specific absorption rate (SAR) due to safety concerns^7^.

The characteristics of the materials used to fabricate the antenna impact its final parameters and are crucial to how well it performs. In principle, the utilized material for the antenna substrate influences its outcomes, including gain and total efficiency^8^. Plastics, paper, textiles, bio-polymers, and Tencel fabric are bio-degradable materials suitable for antenna substrates^9,10^. Moreover, Rogers printed circuit boards (PCBs) are broadly used as antenna substrates due to their high dielectric constants and temperature stability^11^.

De Cos et al. presented an aluminum-metalized polypropylene (PP) low-cost, flexible antenna manufactured by laser micromachining^12^. Their proposed antenna resonated at dual-band radiofrequency (2.45 GHz and 5.8 GHz), which is suitable for wearable applications. Haraz et al.^13^ designed, fabricated, and tested three printed log periodic dipole array (PLPDA) antenna prototypes resonating at the v-band frequency range. The performance of the proposed antennas was compared in terms of gain, bandwidth, radiation pattern, side-lobe suppression, and front-to-back ratio. Bendaoudi et al.^14^ studied the effect of using different dielectric substrates, including Bakelite, Rogers, RO 4232, and RT Duroid, on the acquired antennas’ parameters (i.e., reflection coefficient, bandwidth, directivity, and radiation pattern).

Laser-induced breakdown spectroscopy (LIBS) is a straightforward, fast, and quasi-nondestructive spectrochemical analytical technique used for elemental analysis of different materials. LIBS has been used in numerous applications, including industrial^15^, metrological^16^, medical^17^, and biological^18^, in addition to adulteration detection and classifications of food products^19–22^, to mention some. It has also been used to discriminate between different types of toners employed in laser printers and photocopiers^23^. The typical LIBS system consists of a pulsed laser source (nanosecond, picosecond, or femtosecond), which produces a plasma plume upon focusing the laser light onto the sample under investigation, and a spectrometer–detector system that disperses the collected plasma emission light that is converted to a displayable spectrum. The obtained spectrum presents the spectral lines corresponding to the specific elements existing in the studied material^24,25^. Moreover, statistical multivariate chemometric methods such as principle component analyses (PCA) and artificial neural networks (ANN) are usually integrated with the LIBS technique to simplify the acquired data sets^26,27^.

The current contribution examines the impact of altering the substrate utilized in antenna fabrication on the antenna’s performance. The same antenna design was implemented using two different substrate materials (Rogers). The performance of the designed antennas was tested considering adipose tissue (i.e., fat) as the surrounding medium. The elemental analysis of the two distinct Rogers (RO 3210 and RO 4003) was determined via LIBS. Additionally, partial least square regression and principle component analysis techniques were used to assess the recorded LIBS spectra. Utilizing the two Rogers, two antennas of the same design were simulated and manufactured. Their reflection coefficients (S11) and other parameters such as gain, radiation patterns, voltage standing wave ratio, and efficiency were compared.

## Materials and methods

### Antenna design

The first step in antenna design is to define the surrounding medium. In our case, we assumed that the proposed antennas are implanted in adipose fat tissue. The electromagnetic (EM) characteristics of this tissue are dispersive. Table 1 summarizes fat tissue characteristics at 405 MHz^28^.

**Table 1:** Fat parameters at the resonance frequency (405 MHz).

Tissue	Conductivity	Relative permittivity	Loss tangent	Wavelength	Penetration depth
Fat	0.102 S/m	5.28	0.14	0.054 m	0.11 m

Compact structure, small size, low weight, and simple processing are benefits of the proposed antenna’s meander structure. The implanted antenna with a flat meander structure is a good option when combined with the spatial structure of the implantable electronic device. The bus length will impact the resonance characteristics of the antenna during the design phase. The route of the current will be extended as the lengthening increases, shifting the resonance frequency to lower frequencies. The following equation presents the antenna bus calculation formula;

1 $${\text{L}}=\frac{{\text{c}}}{4{\text{f}}\sqrt{{\upvarepsilon }_{{\text{r}}}}}$$

where *L* is the bus length of the antenna, *f* is the antenna’s resonant frequency, ${\upvarepsilon }_{{\text{r}}}$ is the dielectric constant of the substrate, and *c* is the speed of electromagnetic waves in free space.

The environment of the proposed implantable antenna is fat rather than free space. Accordingly, the effective dielectric constant is related to the dielectric constant and volume fraction of fat media. After analyzing the parameters, ${\upvarepsilon }_{{\text{r}}}$ changes to ${\upvarepsilon }_{{\text{eff}}}$ (the effective permittivity of homogenized dielectric mixtures). The dielectric constant obtained in this way will be more accurate according to Lichtenecker’s formula^29^:

2 $${\text{ln}}\left({\varepsilon }_{eff}\right)= {v}_{1}{\text{ln}}\left({\varepsilon }_{1}\right)+ {v}_{2}{\text{ln}}({\varepsilon }_{2})$$

where *ε*_1_ is the homogeneous medium’s permittivity (i.e., fat tissue) and *ε*_2_ is the permittivity of the substrate material (i.e., Roger). ${{\text{v}}}_{1}$, ${{\text{v}}}_{2}$ are the relative volume fractions of the two substances (i.e., fat and Roger).

**Figure 1:**
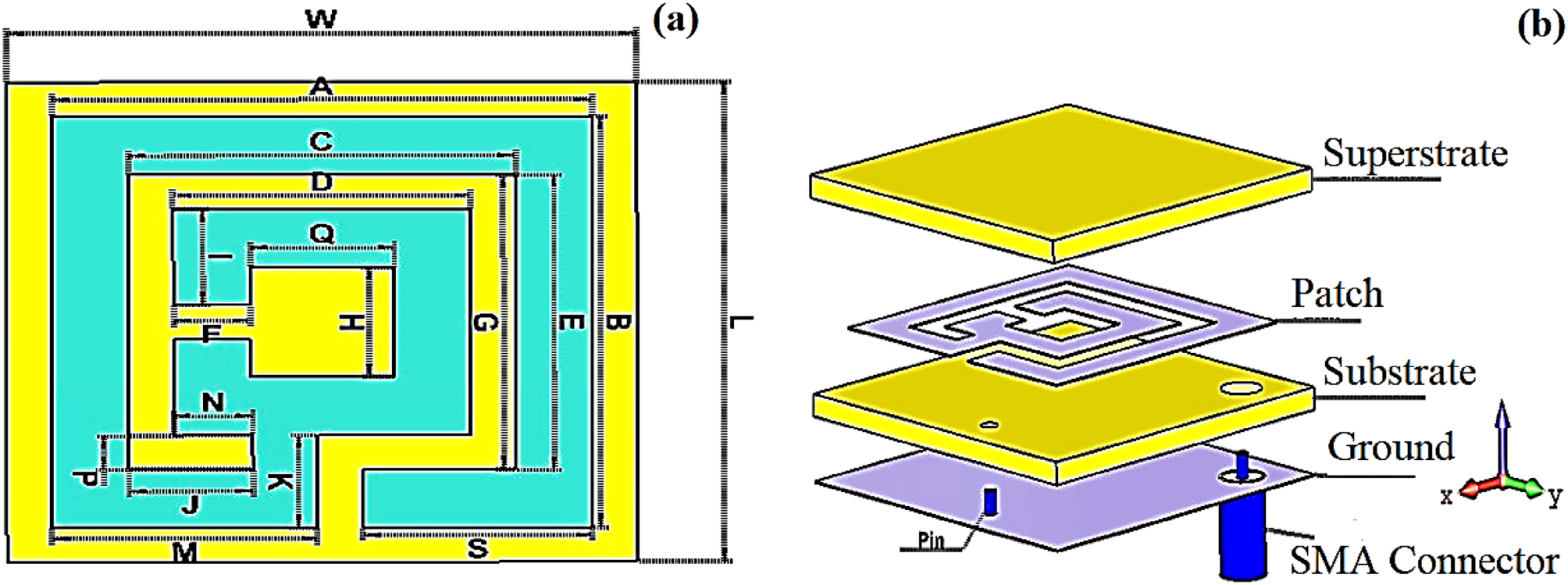
The proposed antenna configuration and dimensions.

The dimensions of the proposed antenna concerning the resonant frequency are defined by 0.116 λ_0_ × 0.116 λ_0_ × 0.006 λ_0_. The design process is performed according to the primary design of the meander patch antenna, as presented in Fig. 1. Moreover, the dimensions of the proposed antenna are summarized in Table 2. CST simulation package is used to design the proposed antenna. The feed point was chosen to guarantee the 50 Ohm input impedance. The suggested antenna has three layers: the substrate (i.e., Roger), the groundsheet, and the patch (i.e., copper). The substrate is the first layer connected to the patch via a pin. A superstrate is applied to permit the use of the antenna on additional tissue measurements and prevent the shorting between patch and substrate, preventing functioning as a patch radiating element. Manufacturing and testing were carried out at the National Telecommunication Institute (NTI) in Cairo, Egypt.

**Table 2:** Dimensions of the proposed antenna (in mm).

A	B	C	D	E	F	G	H	i	J	K	L	M	N	Q	S	W
12	12	9	6.6	9	2	7	3	3	3	3	14	6	2	3	5	14

The thickness of the two types of Rogers (RO 3210 and RO 4003) tested and used in the antenna’s design was almost the same (~ 0.8 mm). The EM characteristics of the substrate and superstrate at 405 MHz are shown in Table 3.

**Table 3:** EM characteristics of the utilized Rogers substrates.

Substrate	${\varepsilon }_{r}$	${\mu }_{r}$	$tantan \delta$
RO 3210	10.2	1	0.0027
RO 4003	3.38	1	0.0027

#### Current distribution

**Figure 2:**
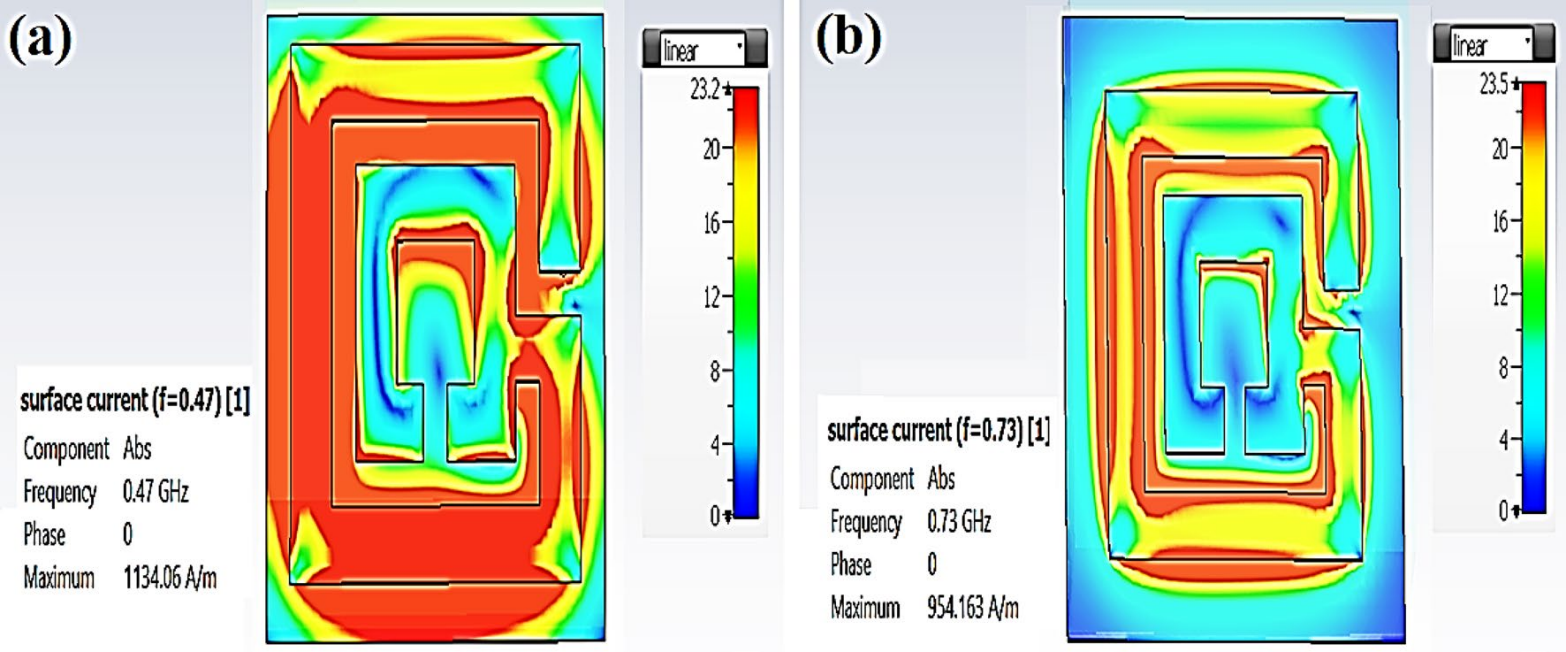
Current distribution of the proposed antenna at MICS band (**a**) RO 3210, (**b**) RO 4003.

The proposed antenna’s surface current distribution was obtained to demonstrate the operation mechanism of the two proposed Rogers types (see Fig. 2). The current distribution is antenna structure dependent on producing the dip at the resonance frequency for both RO types, as the current concentration all over the antenna surface.

### Laser-induced breakdown spectroscopy (LIBS)

The LIBS standard experimental setup used in this study was documented in detail elsewhere^30^. The incident laser source used to generate plasma onto the surface of the Roger substrate) is a Q-switched Nd:YAG laser (Brio, Quantel, France) with λ = 1064 nm and 50 mJ pulse energy. The laser beam was focused onto the sample surface using a planoconvex lens (5 cm focal length). The distance from the lens to the sample surface was adjusted via an X–Y–Z micrometric translational stage. The light emitted from the plasma was collected using a 2-m fused silica optical fiber (600 µm core diameter) and coupled with an echelle spectrometer (Mechelle 7500, Multichannel, Sweden). The spectrometer has a built-in ICCD camera (DiCAM PRO, PCO-Computer optics, Germany) to detect the dispersed plasma emission light. The optimized optical trigger delay time of the ICCD was 1000 ns, and the gate width was 1500 ns. The LIBS++ software was used to conduct the spectral analysis^31^.

### Principle component analysis (PCA)

PCA is a statistical multivariate analytical approach commonly used to reduce the number of variables and simplify the representation of the sample distribution. Such an approach can envisage complex multivariate data on a low-dimensional scale, where the suggested dimensionality reduction preserves most of the variance included in the original data. This chemometric method reduces the original data set’s dimensionality by generalizing the native variance by transforming the original high-dimensional space (wavelength as a variable in LIBS) into a smaller set of independent principal components^32,33^.

In LIBS, PCA effectively decreased the dimensionality of the measured data set, represented in the collected spectra, with the least possible loss of crucial information^34^. The principle components were initially calculated using a mean vector, and a mean-adjusted matrix was produced. This mean matrix creates a covariance matrix that contains the redundant data. The principal components are then determined by computing the covariance matrix’s eigenvectors. The initial variables are essentially combined linearly to form the principal components. They are designed so that the first principal component (PC1) explains the possible variance. The next most significant variance is explained by the second principal component (PC2), which is parallel to PC1 and so on^35^. In the current implementation, the PCA analysis was performed using a commercial software package (OriginPro2018, SR1 b9.5) with 25 LIBS spectra per sample. Every spectrum is the average of five spectra collected from different positions. Consequently, the redundant information in the LIBS spectra is reduced, and the Roger types can be distinguished.

### Partial least square regression (PLSR)

PLS is used in the present study to find the correlation between the obtained repeated spectral measurements. By developing new predictors known as “components,” PLSR is used to model the response variable of datasets with a high degree of predictor correlation^36^. Unlike PCR, the response variable was considered when building the PLSR components, which show the observed variability in the predictors^37,38^. PLSR is typically used to identify the best line that fits the dataset. As a result, it is ensured that the measured data is correlated and that no unusual values exist^39^. The current study has implemented PLSR of the acquired LIF spectra via a Matlab function called “plsregress”. A PLSR of the response matrix on the predictor variables matrixes was conducted after determining the PLS component count, and the predictor and response loadings were then recovered. The response matrix in the current implementation includes the intensities corresponding to each wavelength over the whole LIBS spectrum.

## Results and discussion

### Elemental analysis of the Rogers types via LIBS

**Figure 3:**
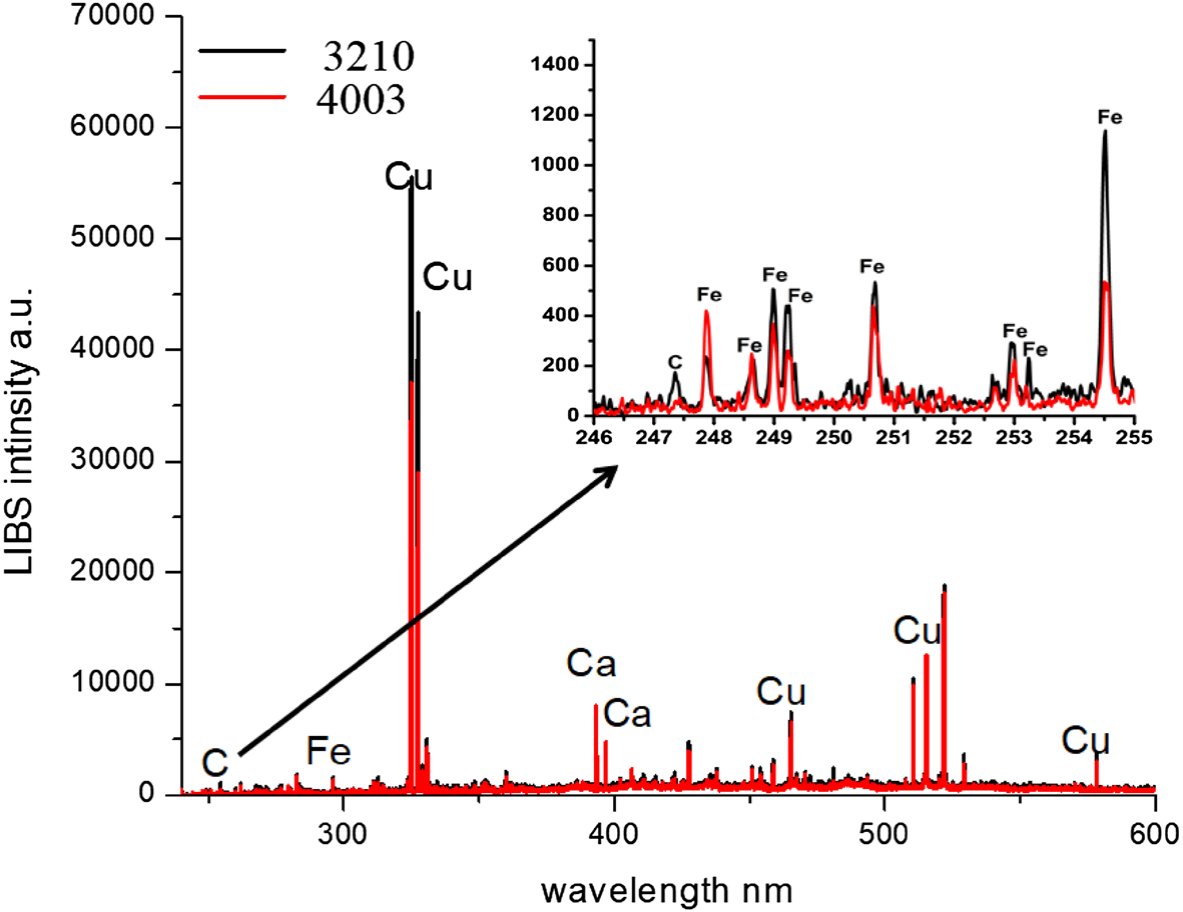
LIBS spectra for the two Rogers, the inset spectra show the Fe spectral lines in the UV wavelength range.

Figure 3 shows the LIBS spectra of the Rogers (RO 3210 and RO 4003). Each spectrum is an average of 25 spectra for every type. It is evident from the figure that there is no significant difference between the two Rogers types’ spectra, namely in the copper (Cu) spectral line at 324.74 and 327.39 nm. Moreover, the carbon line at 247.8 nm and the iron lines (Fe) at 259.9, 273.2, and 274.9 nm show up clearly in both cases of the Rogers spectrum.

It wasn’t easy to distinguish between the spectra of RO3210 and RO4003. However, copper and iron lines can be detected in both of these Rogers’ spectra, which can be identified by careful examination. The enlarged spectra in Fig. 3 show these unique spectral lines. This observation demonstrates the potential of LIBS for (partial) separation between the several Rogers by identifying spectral lines that can be utilized as fingerprints in their emission spectra.

### Statistical analysis using PCA

**Figure 4:**
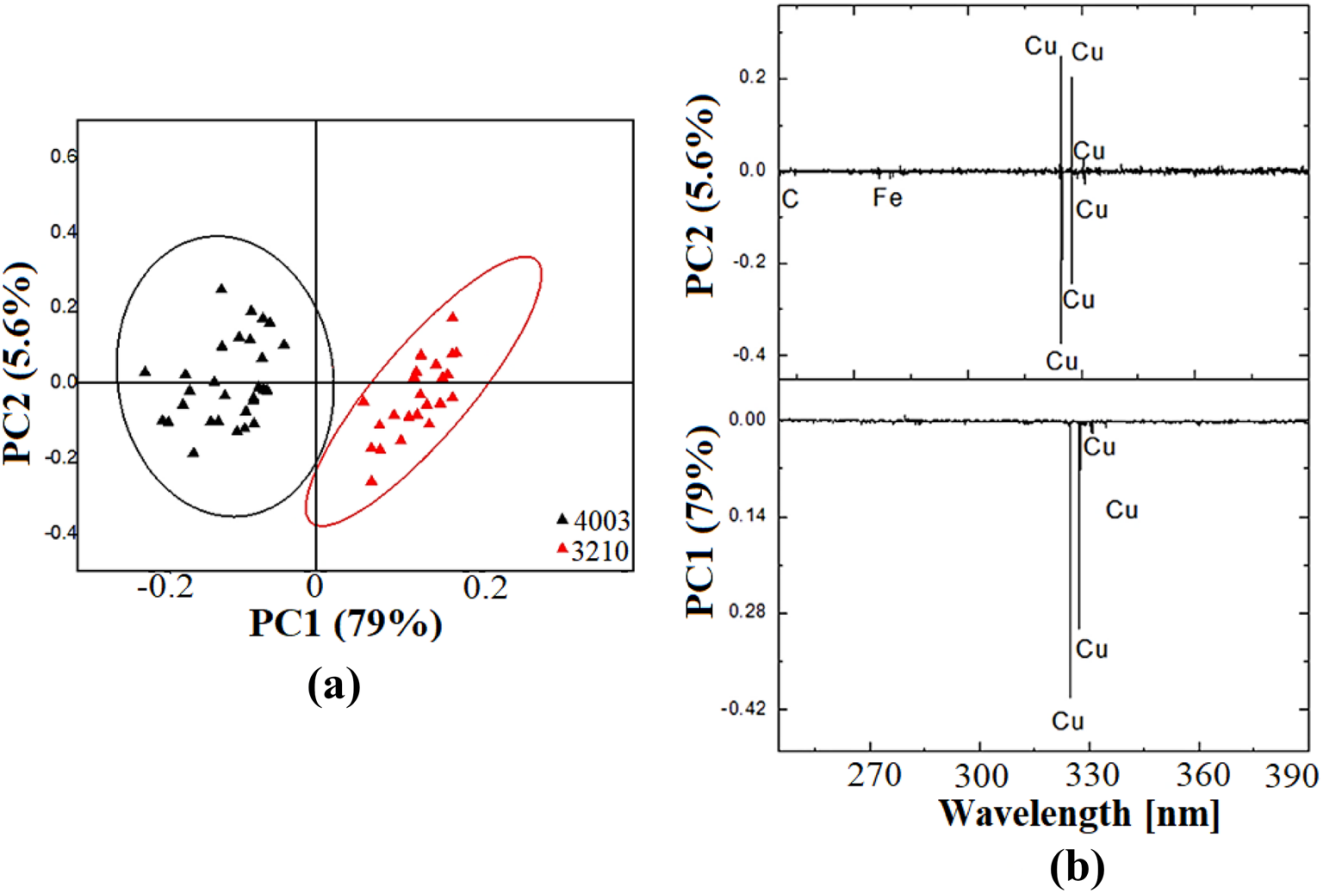
(**a**) The score plots from PCA and (**b**) PCA loadings plot on LIBS spectra for discrimination two Rogers.

PCA has been applied to the acquired LIBS spectra to validate the spectroscopic data and help discriminate between different Rogers. The part spectral range (245–390 nm) of each of the 25 spectra for every sample type was used in the analysis. The score plot shown in Fig. 4a allows good discrimination between the Rogers of the two types. PC1 and PC2 account for 84.6% of the data variance, with PC1 = 79.0% and PC2 = 5.6%.

The corresponding loadings plots depicted in Fig. 4b show that Cu content majorly contributed to the discrimination success, followed by differences in the Fe and C content. PC1 shows the significant contribution of Cu content to the PCA discrimination. It can be seen that C, Fe, and Cu are in the negative part of PC2, which contributes to the 4003 Rogers, as shown in Fig. 4a. Concerning PC2 in the same wavelength range, there is a significant contribution of Fe and C in the negative part, which are substantial elements in the 3210 Rogers. The presence of Fe and C as minor elements in the negative part of PC1 in the same wavelength range is relevant to the 3210 Rogers.

### Measurements via PLS

**Figure 5:**
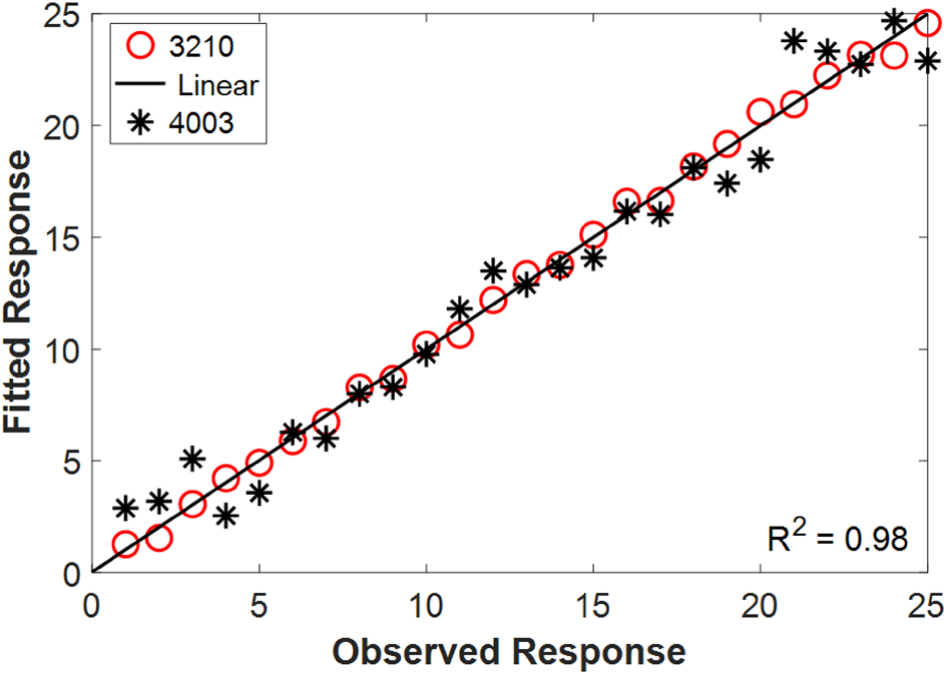
Partial Least Squares Regression (PLSR) of the obtained LIBS spectra using the two Rogers.

Through partial least-squares regression, the correlation of the experimental measurements was assessed. As a result, the obtained coefficient of determination (i.e., R-squared value) was 0.98, as illustrated in Fig. 5. The loaded data in the PLSR model were the spectral intensities of 25 samples of RO 3210 and RO 4003, respectively.

### Antenna parameters

**Figure 6:**
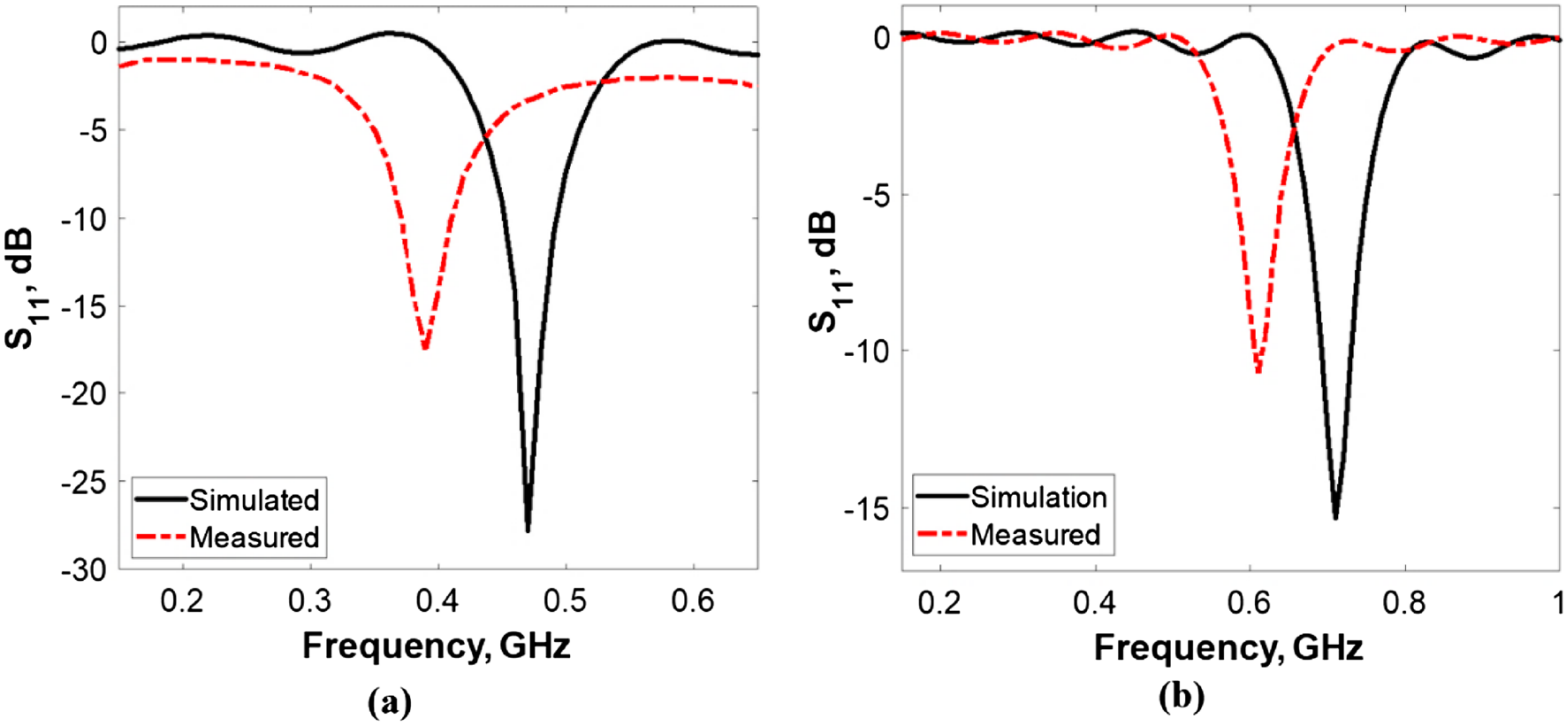
The S11 for the two proposed Rogers (**a**) RO 3210, (**b**) RO 4003.

The simulated and measured reflection coefficient (S11) for the two investigated antennas is presented in Fig. 6. A network Analyzer (R&S, ZVB, Rohde and Schwarz, USA, 10 MHz–20 GHz) was used to measure S11 of the fabricated antennas. For RO 3210, the simulated S11 was − 27 dB at 0.47 GHz, while the measured S11 was − 17 dB at 0.40 GHz. While using RO 4003, the measured S11 was − 10 dB at 0.61 GHz, and the simulated was − 15 dB at 0.73 GHz. The shift in the resonance frequency between simulations and measurements is common and occurs due to imperfections and manufacturing tolerances. Additionally, fatty tissue demonstrates loss and dispersion properties, attenuating and absorbing electromagnetic waves at various frequencies. Significant variations from the simulated findings can occur in the resonance frequency due to these losses and dispersion effects^40,41^.

**Figure 7:**
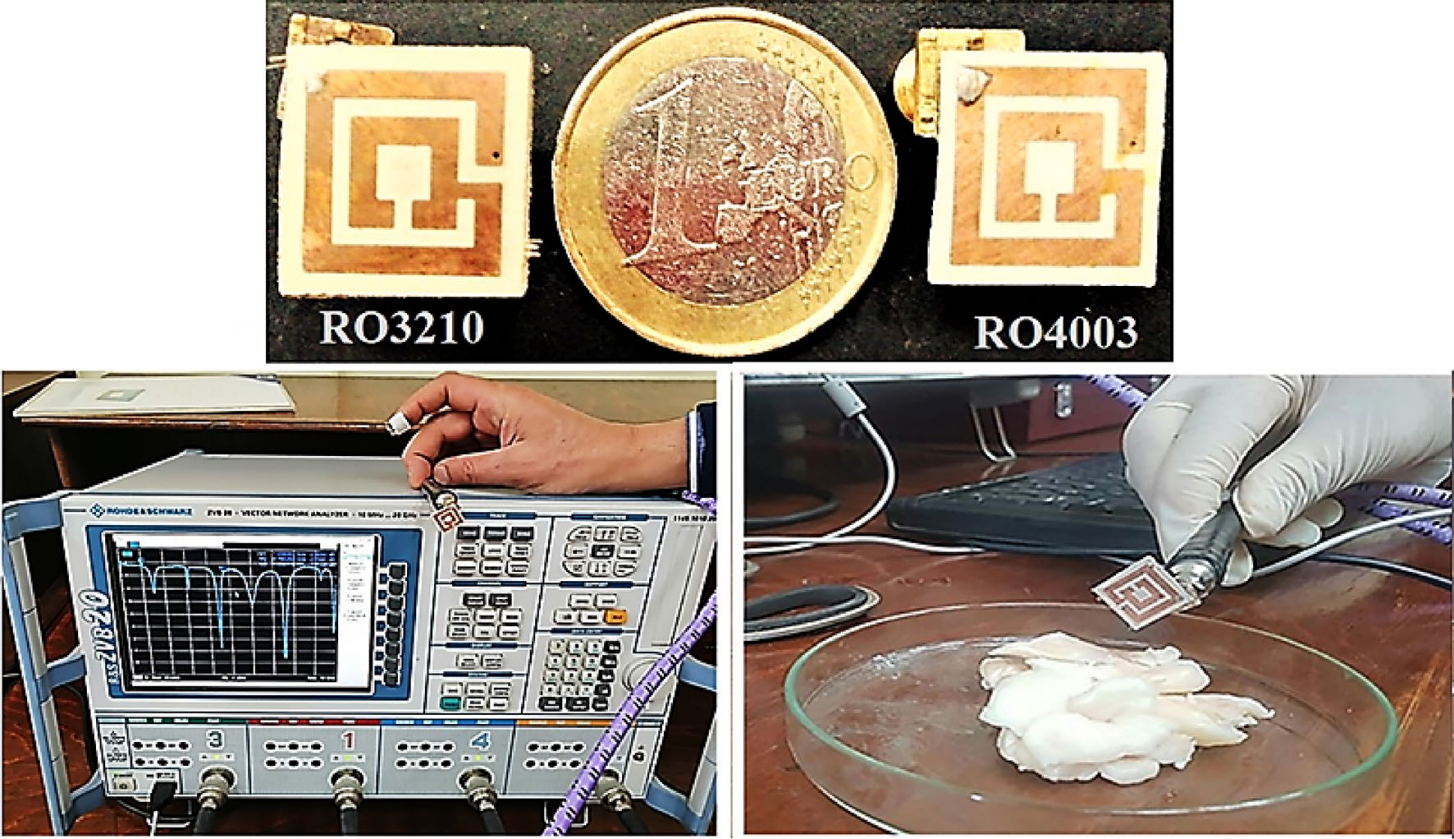
Photographs of the two manufactured antennas and the measurement process after adding the examined tissue.

As resonant circuits represent Rogers, any increase in the copper content affects the resonance frequency, leading to a frequency shift. It is evident from Fig. 6 that changing the Rogers type resulted in a shift in the resonant frequency due to the dielectric constant change. The manufactured antennas and the measuring process where the entire antenna has been embedded in adipose fat tissue are shown in Fig. 7.

**Figure 8:**
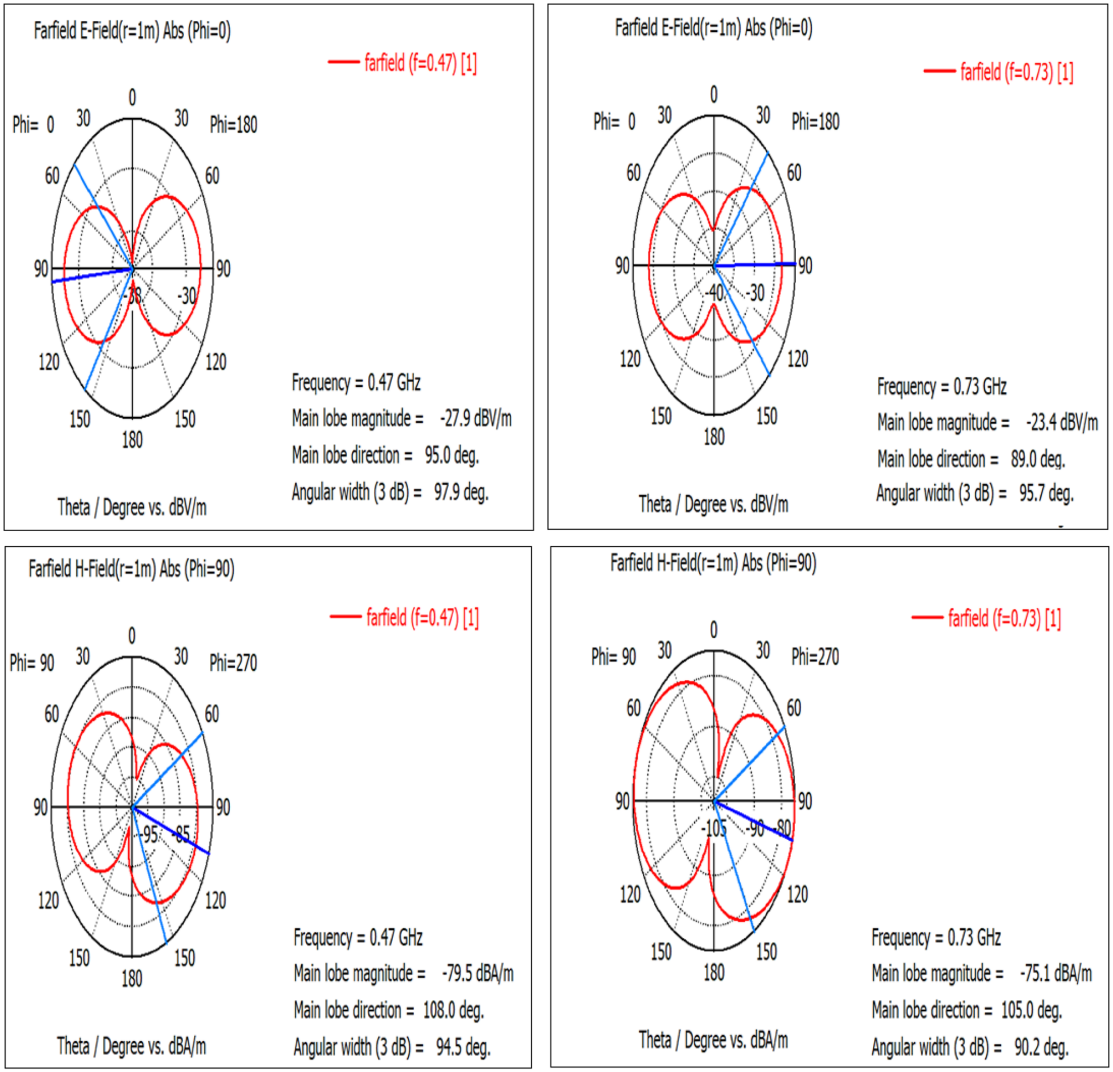
The radiation pattern of the E-plane at φ = 0° (**a**) RO 3210, (**b**) RO 4003 and of the H-plane at φ = 90°, (**c**) RO 3210, (**d**) RO 4003.

The E-plane of the radiation pattern of the proposed antenna (E_θ_ at φ = 0°) and the H-plane radiation pattern (E_θ_ at φ = 90°) are presented in Fig. 8. Both the E- and H-plans radiation patterns demonstrate that the proposed antennas radiate bidirectional in both cases (RO 4003) and (RO 3210).

Specific Absorption Rate (SAR) is a measurement of the amount of transmitted RF energy that is absorbed by the tissue. It depends on the tissue’s mass density $\rho$ in kg/m^3^, electrical conductivity $\sigma$ in S/m, and the E-field in V/m created by the radiated energy. The SAR is calculated by averaging (or integrating) over a specific weight (typically 1 g or 10 g), as illustrated in Eq. (1).

3 $$ SAR = \int_{sample} {\frac{{\sigma (r)\left\lceil {E(r)} \right\rceil^{2} }}{\rho (r)}\;dr} $$

**Figure 9:**
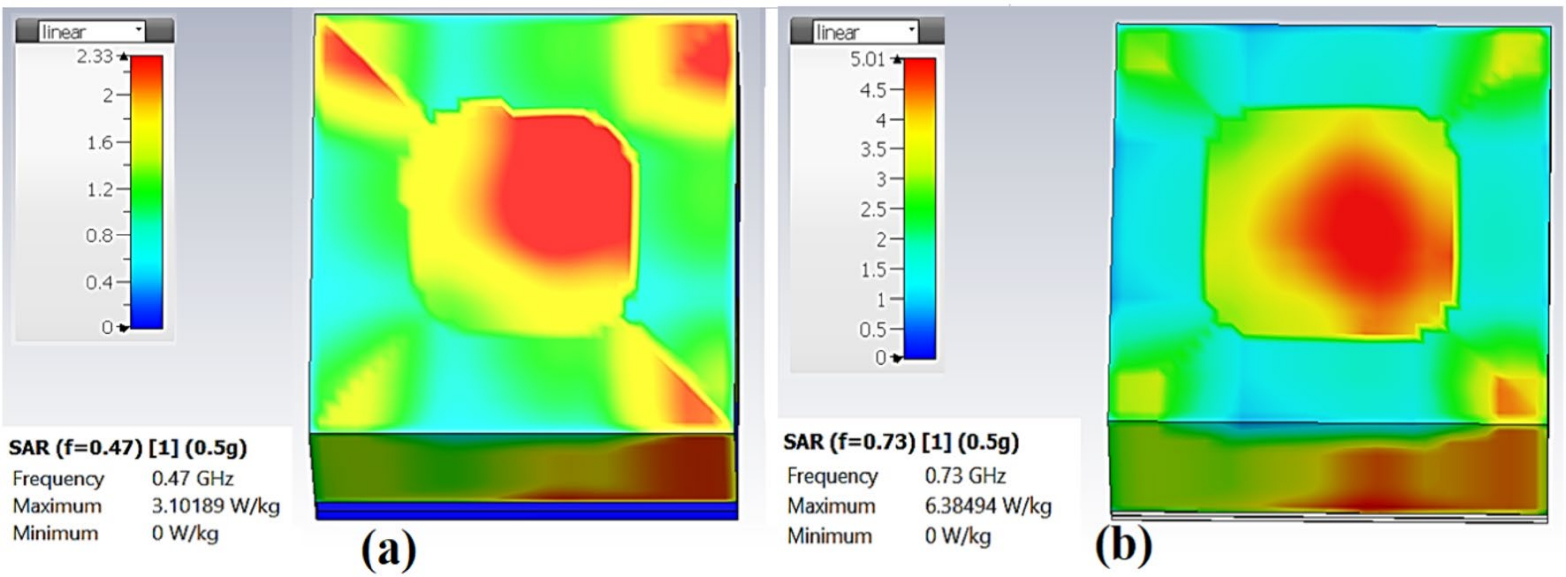
SAR for the proposed antenna (**a**) Rogers 3210, (**b**) Rogers 4003.

SAR is determined by averaging (or integrating) over a predetermined weight. Figure 9 illustrates the simulated SAR value upon insertion into fat tissue. The 0.7 g’s maximal SARs are 8.8 W/kg for RO 3210 and 1.94 W/kg for RO 4003. SAR enhances the main lobe and reduces the back lobe, i.e., a small amount of electromagnetic waves is radiated to the tissue.

**Figure 10:**
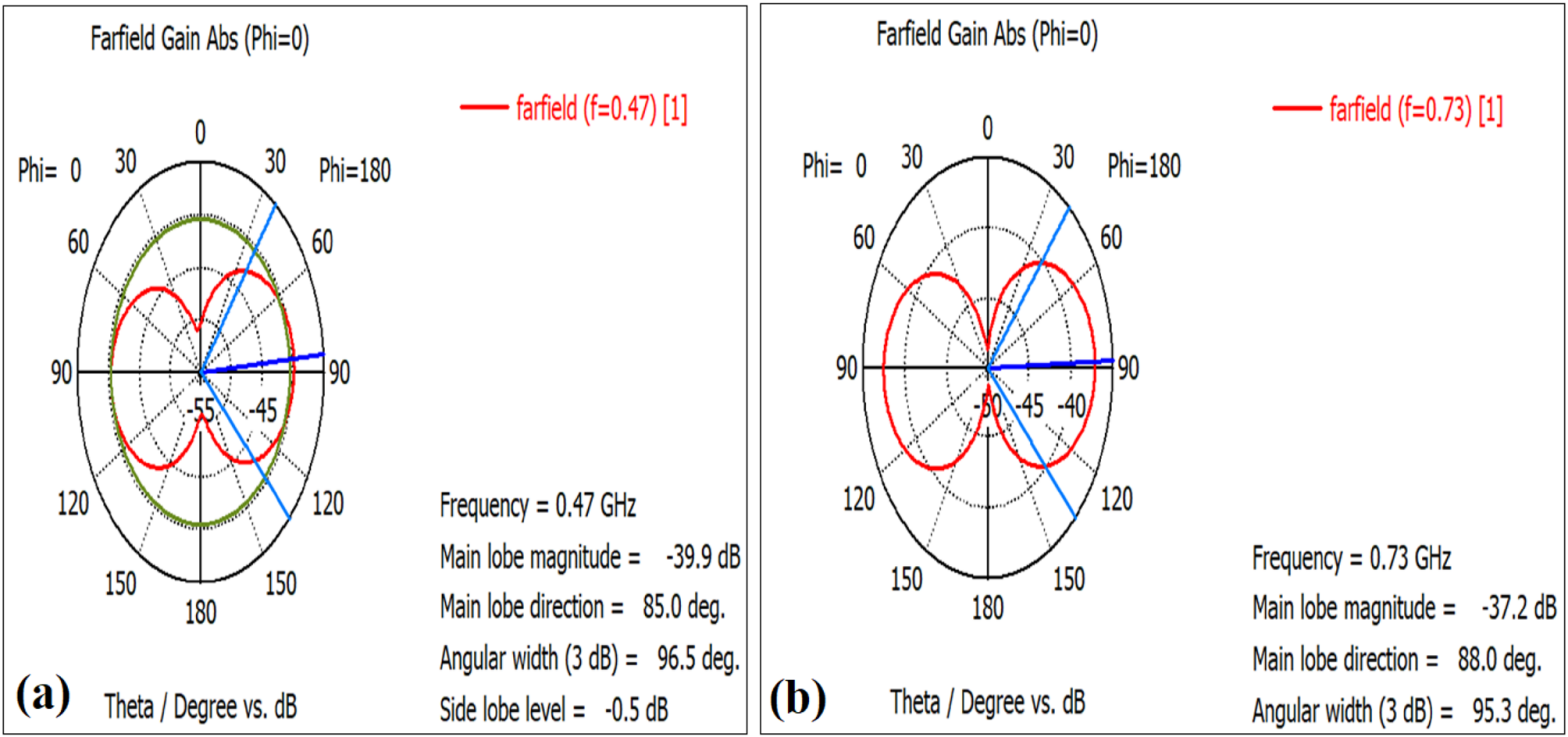
The proposed antenna’s gain (**a**) Rogers 4003, (**b**) Rogers 3210.

The acquired gain of the proposed antenna for the Rogers (3210, 4003) types is presented in Fig. 10. The Gain was − 37 dB in the case of using Rogers 4003 as substrate and − 40 dB in the case using Rogers 3210. The discrepancy may be due to the variation in the dielectric constant of the two Rogers.

Table 4 summarizes the main parameters of the two designed antennas, including resonance frequency, reflection coefficient gain, SAR, and bandwidth.

**Table 4:** Comparison of the parameters of the proposed antennas loaded by the two Rogers.

Antenna	Resonance freq. (GHz)		S11 (dB)		Bandwidth (KHz)		SAR (W/kg)	Gain (dB)
	Simulated	Measured	Simulated	Measured	Simulated	Measured		
RO 4003	730	610	− 15	− 9	142	133	1.94	− 37
RO 3210	470	402	− 27	− 16	187	300	8.8	− 40

As shown in Table 4, the maximum obtained gain is − 40 dB using Rogers 3210 and − 37 dB for Rogers 4003 at the resonance frequency. This variance is due to changing the Rogers type.

## Conclusion

The present study illustrates the effect of changing the substrate material (Rogers) on antenna performance. Two Rogers substrates were used, namely RO 3210 and RO 4003. LIBS technique was utilized to characterize the raw material of the two Rogers based on the existing elements. The two Rogers spectra had no notable difference, especially in the copper contents. However, carefully examining these Rogers’ two spectra reveals that carbon and iron lines distinguish them. The two Rogers were used to simulate and manufacture two antennas with an identical design. The antenna performance was studied and compared in terms of reflection coefficient, current distribution, gain, radiation pattern, VSWR, and total efficiency upon changing the substrate material. The acquired results revealed a significant role of the substrate material in the antenna performance due to the difference in the dielectric constants.

## Data Availability

The datasets used and/or analyzed during the current study are available from the corresponding author upon reasonable request.
